# Dermatological concerns for women and girls with turner syndrome

**DOI:** 10.3389/fmed.2023.1235187

**Published:** 2023-09-13

**Authors:** David Rodriguez-Buritica, Meaghan Mones, Siddharth K. Prakash, Michelle Rivera, Melissa Aldrich, Megan Rogge, Kate Richardson

**Affiliations:** ^1^Department of Pediatrics, Division of Medical Genetics, McGovern Medical School at the University of Texas Health Science Center at Houston (UTHealth Houston) and Children’s Memorial Hermann Hospital, Houston, TX, United States; ^2^Cancer Center UTHealth Graduate School of Biomedical Sciences, Houston, TX, United States; ^3^Department of Internal Medicine, McGovern Medical School at the University of Texas Health Science Center at Houston (UTHealth Houston), Houston, TX, United States; ^4^Department of Pediatrics, Division of Pediatric Endocrinology, McGovern Medical School at the University of Texas Health Science Center at Houston (UTHealth Houston) and Children’s Memorial Hermann Hospital, Houston, TX, United States; ^5^Brown Foundation Institute of Molecular Medicine, McGovern Medical School at the University of Texas Health Science Center at Houston (UTHealth Houston) and Children’s Memorial Hermann Hospital, Houston, TX, United States; ^6^Department of Dermatology, McGovern Medical School at the University of Texas Health Science Center at Houston (UTHealth Houston) and Children’s Memorial Hermann Hospital, Houston, TX, United States

**Keywords:** turner syndrome, medical dermatology, quality of life, dermatological manifestations, xerosis cutis, keloids, onychodystrophy

## Abstract

**Introduction:**

Turner syndrome (TS) is associated with distinct manifestations in women and girls including short stature, cardiac abnormalities, premature ovarian failure as well as dermatological features, including lymphedema, keloids, onychodystrophy, and acne. Although many dermatological concerns present during the first few decades of life, the overwhelming majority of respondents are not provided with dermatology referrals at diagnosis.

**Methods:**

This cross-sectional study utilized an author designed survey to assess self-reported dermatological manifestations, dermatology referral experience, common therapies for select dermatological conditions, as well as a validated 10-question Dermatology Life Quality Index (DLQI) to assess quality-of-life impact in women and girls with Turner syndrome.

**Results:**

In our cohort, 64% (*n* = 149) had been referred to a dermatologist at some point in their life time. The majority of individuals self-identified their dermatological concern (79.6%) and were referred after a dermatological concern had already occurred (90.2%). The most common dermatological findings reported were xerosis cutis (78.7%), lymphedema (73%), and more than 20 acquired melanocytic nevi (70%). The overall mean DLQI score was 3.52, indicative of a small effect on the patient’s life. Onychodystrophy, history of skin biopsy, and lymphedema were statistically significant to have a higher impact on quality of life.

**Discussion:**

Our data reveal that skin conditions are highly prevalent in the TS population during the early decades of life and affirm utilizing these conditions in the TS diagnostic process, as well as emphasize the need for specialized dermatology referrals to address the detrimental impacts related to skin concerns on quality of life.

## Introduction

Turner syndrome (TS) is a common chromosome condition that affects approximately 64 per 100,000 in newborns (1). The genetic etiology of TS is complete or partial loss of one X chromosome with or without cell-line mosaicism. TS is defined by the presence of distinctive clinical features—the most common being short stature and gonadal failure—in a non-ambiguous female with one or more cell lines with absence of a sex chromosome. In addition to these features, abnormalities of the cardiovascular system, renal system, lymphatic system, dentition, behavior, and skin are common (2–4).

The awareness of dermatological manifestations, especially ones present during early childhood can increase the index of suspicion and lead to an earlier diagnosis, which often remains a problem, in particular for mosaic TS cases. Furthermore, knowledge regarding common features of TS make precise clinical care recommendations possible. For example, endocrinologists utilize guidelines for hormone therapies to promote linear growth, puberty induction, and fertility (5). In contrast, risk stratification and management of dermatological diseases in TS are not outlined in clinical guidelines.

A more recent study conducted by (6) summarized the dermatological findings of 236 individuals with TS at a single clinical site indicating 7.2% had keloids, 71.2% had increased acquired melanocytic nevi (AMN), 2.1% had vitiligo, 3% had alopecia areata, 27.1% experienced lymphedema (ongoing or resolved), 3% had acne vulgaris, and 2.1% had basal cell carcinoma. Nail abnormalities manifesting as small, hyperconvex, pitted, or otherwise dysplastic nails are present in 80% of women with TS (3). Lastly, growth hormone (GH) therapy has been suggested to increase the growth rate of AMN, but this is not universally accepted (7, 8).

The current clinical guidelines lack tangible information about the appropriateness and frequency of dermatological surveillance and specialty referrals for women and girls with TS (9). Despite the increased prevalence of numerous dermatological diseases, there is a general lack of guidance for practitioners that care for individuals with TS. Moreover, there are currently no studies that investigate the quality-of-life impact that these common skin manifestations have on this population. Thus, it is pertinent to further characterized dermatological diseases in TS and provide concrete recommendations to guide physicians who care for affected women and girls. In this study, we aim to describe the occurrence of self-reported dermatological concerns in women and girls with TS and to assess the impact on their health perception.

## Materials and methods

### Study design and population

This cross-sectional study was designed and approved under the Institutional Review Board of University of Texas Health Science Center at Houston (HSC-MS-15-0120) to assess self-reported dermatological manifestations, dermatology referral experience, common therapies for select dermatological conditions, and quality-of-life impact in women and girls with Turner syndrome at any age.

Our study population included any female with a clinical and genetic confirmation of Turner syndrome. Respondents who were less than 18 years old required the survey be completed by a parent or relative. Our recruitment strategy included contacting the following groups: members of the UTHealth Turner Syndrome Research Registry, members of the Turner Syndrome Society of the United States (TSSUS), and members of Turner syndrome social media outlets (i.e., Facebook groups). Our questionnaire was distributed in three different ways. For the UTHealth registry, members were contacted by both email and phone call to complete our electronic survey either independently or assisted over the phone. Members of the TSSUS were contacted through the moderated monthly newsletter and social media outlets with an anonymous Qualtrics survey link (Qualtrics, Provo, UT). Facebook group administrators were contacted and consented before posting an anonymous survey link. Surveys distributed to the Facebook groups and TSSUS included exclusionary questions at the start of the survey to identify repeat respondents.

### Questionnaire design

The questionnaire was developed after an extensive literature review and consisted of author-designed questions regarding demographic information, referral experience, dermatological manifestations, treatment plans, and the validated Dermatology Life Quality Index (DLQI) (10). The demographics section included type of respondent (person with TS or parent), age range, ethnicity, and karyotype, if known. The referral experience section queried whether they have ever been referred to see a dermatologist, who referred them, and if they desire a referral now. The quality-of-life scale was a validated 10-question Dermatology Life Quality Index (DLQI). The dermatological manifestations section included inquiries about dry skin, lymphedema, hair loss, dysplastic nails, >20 skin nevi (referring to AMN), red marks or patches, skin biopsies, skin cancer, vitiligo, alopecia, keloids, scarring, and acne. All questions if selected “yes” included additional inquiries about age of manifestation. Some questions if selected “yes” included additional inquiries about treatment plan, area of body affected, or relevant secondary manifestations. In addition, all participants were asked if they have taken growth hormone and if they have concerns about side effects like increased number of AMN, hirsutism, and swelling. Lastly, each participant was asked to indicate what manifestations asked about in the survey are present in a first-degree relative, as well as write any other concerns they may have outside of those asked in the survey.

### Statistical analysis

All analyzes were performed using Stata v.11. Descriptive analysis was executed for all variables (frequencies, median, and SD, as applicable). For statistical comparisons of significance, *Z*-test proportion tests, T-tests, Chi-Squared analysis, and Fischer’s exact analyzes were used with a value of *p* of <0.05. To evaluate the heterogeneity of the data across the three groups, multivariate test of means was completed with a value of *p* of <0.05.

## Results

### Demographics

We received a total of 241 surveys with 89.2% (*n* = 215) fully completed while the remaining 10.8% (*n* = 26) were partially completed. Our sample consisted of 191 responders who had a personal history of TS (80.6%), while the remaining respondents were completed by a parent or guardian. Demographics and karyotypes of our survey population can be seen in Tables 1, 2, respectively.

**Table 1:** Demographic characteristics.

Age range (*n* = 236)	*n* (%)	Race (*n* = 234)	*n* (%)
0–10	2 (0.9%)	American Indian or Alaska Native	4 (1.7%)
10–14	19 (8.1%)	Asian	3 (1.3%)
14–18	14 (5.9%)	Black or African American	3 (1.3%)
19–24	31 (13.1%)	Native Hawaiian or Other Pacific Islander	0
25–34	45 (19.1%)	White	216 (92.3%)
35–44	52 (22.0%)	Other	8 (3.4%)
45–54	38 (16.1%)		
55+	35 (14.8%)		
Ethnicity (*n* = 229)	*n* (%)	Participant (*n* = 237)	n (%)
Hispanic or Latino	20 (8.7%)	A person with TS	191 (80.6%)
Not Hispanic or Latino	209 (91.3%)	The parent or guardian of a person with TS	46 (19.4%)
		Someone else	0

Demographic information of subjects with TS in our cohort.

**Table 2:** Self-reported blood karyotype.

Karyotype (*n* = 165)	*n* (%)
45,X	75 (45.4%)
45,X/46,XX	25 (15.2%)
45,X/46,XY	4 (2.4%)
Deletion Xp	5 (3.0%)
Isochromosome	10 (6.1%)
Mosaic with ring	15 (9.1%)
Mosaic with 47,XXX	15 (9.1%)
Other mosaic with Y chromosome	5 (3.0%)
Something else	11 (6.7%)

Percentage breakdown of self-reported karyotypes for individuals with TS in our cohort.

### Referral experience

Our survey revealed that 149 individuals (64.0%) have been referred to see a dermatologist at some point in their lifetime. Of those respondents, 47 (33.1%) were referred by a medical provider administering TS care, 14 (9.8%) were referred at the time of TS diagnosis, and 113 (79.6%) self-identified their dermatological concern. Regardless of referral history, 129 (57.6%) of our sample reported that they are currently concerned about their skin and 52 individuals (23.2%) currently want a dermatology referral (Table 3).

**Table 3:** Self-reported dermatology referral experience.

Referral status (*n* = 233)	*n* (%)	Referred by (*n* = 142)	*n* (%)	Referral timing (*n* = 143)	*n* (%)
I have been referred	149 (64.0%)	My doctor that provides TS care	47 (33.1%)	At the time of my diagnosis	6 (4.2%)
I have not been referred	84 (36.0%)	Somebody else	95 (66.9%)	After a problem came up	129 (90.2%)
				Both	8 (5.6%)
Who noticed (*n* = 142)	*n* (%)	Currently concerned (n = 224)	*n* (%)	Want referral (*n* = 224)	*n* (%)
My doctor noticed a problem	29 (20.4%)	I am currently concerned about my skin	129 (57.6%)	Yes	52 (23.2%)
I noticed a problem	113 (79.6%)	I am not currently concerned about my skin	95 (42.4%)	No	172 (76.8%)

Analysis of past referral experience to dermatology from our TS cohort.

### Dermatological manifestations

#### Xerosis cutis

One-hundred and seventy-four (78.7%) respondents reported a personal history of dry, flaky, or scaly skin. Of this group, the median age of primary onset was in the second decade of life. Ninety-two (64.8%) of respondents with a history of dry skin also reported a positive family history. See Table 4 for complete data on frequency, median age of onset, and positive family history.

**Table 4:** Self-reported dermatology manifestations.

	*n* (%)	Median age of onset (age range)	Positive family history *n* (%)
Xerosis cutis	174 (78.7%)**	11–20 years old	92 (64.8%)
Lymphedema	73 (33.0%)**	0–2 years old	21 (36.8%)
Hair Thinning	83 (37.6%)**	21–30 years old	35 (57.4%)
Onychodystrophy	95 (43.4%)	0–10 years old	17 (26.6%)
>20 skin nevi (AMN)	154 (70.0%)**	0–10 years old	76 (59.8%)
Skin cancer	19 (8.8%)**	31–40 years old	8 (47.1%)
*Have you had a skin biopsy?*	84 (38.2%)	21–30 years old	38 (58.5%)
*Was the area sun exposed?*	16 (84.2%)		
Vitiligo	13 (6.1%)**	11–20 years old	3 (37.5%)
Alopecia Areata	15 (7.0%)**	21–30 years old	4 (40.0%)
Keloids	88 (40.9%)**	11–20 years old	24 (40.7%)
Acne	62 (28.7%)**	11–20 years old	26 (51.0%)
*Have you taken progesterone?*	46 (74.2%)		
		*N* (%)	
Have you ever taken growth hormone (GH)?		144 (66.7%)	
Do you have concerns about...			
GH and increased number of skin moles?		34 (23.8%)	
GH and excess hair growth?		28 (19.4%)	
GH and swelling?		34 (23.8%)	

Percentage breakdown of each dermatological manifestation with median age of onset and positive family history.

#### Lymphedema

Lymphedema was defined as excess fluid collecting in tissues causing swelling, and 73 (33.0%) respondents reported a positive personal history. The median age of onset was between birth and 2 years of age consistent with the known natural history of primary lymphedema in TS. *Z*-test proportion analysis demonstrated a statistically significant (*p* < 0.05) increase in prevalence of lymphedema in this sample compared to the general population prevalence of 0.1%, and a statistically significant (*p* < 0.05) lower prevalence compared to the previously estimated 66.7% overall lifetime frequency in the TS community. Fischer’s exact analysis demonstrated an association between a higher prevalence of primary lymphedema and 45,X karyotype.

#### Hair thinning

Eighty-three (37.6%) respondents reported a history of hair thinning. The median age of onset was within the third decade of life. Of these respondents, 35 (57.4%) reported a family history of hair thinning.

#### Alopecia areata

Alopecia areata was defined as sudden and localized hair loss resulting in baldness, and 15 (7.0%) respondents reported a positive personal history. The median age of onset was in the third decade of life. Of this, four (40.0%) reported a family history of alopecia. The most common treatments reported were corticosteroid injections (*n* = 6, 40.0%), cosmetic replacements (*n* = 4, 26.7%), and no treatment (*n* = 4, 26.8%). *Z*-test proportion analysis showed a significantly (*p* < 0.05) higher prevalence of alopecia areata in this sample compared to the general population prevalence estimated up to 2% (11).

#### Onychodystrophy

Onychodystrophy was defined as small, pitted, abnormally shaped, and/or painful nails. Approximately 44% (n = 95) reported a positive personal history with a median age of onset within the first decade of life. Seventeen (26.6%) reported a positive family history.

#### Acquired melanocytic nevi (more than 20)

One-hundred and fifty-four (70.0%) of our sample reported the presence of more than 20 AMN at any point during their lifetime. The median age of onset was within the first decade of life. Seventy-six (59.8%) reported a positive family history. More than 20 AMN was significantly associated with both a history of skin cancer and skin biopsy (*p* < 0.05). The specific type of skin cancer was not recorded.

#### Skin cancer

Nineteen (8.8%) respondents reported a personal history of skin cancer. Sixteen (84.2%) reported the skin cancer arose in a sun-exposed area. The most affected anatomical region was the face (*n* = 12, 63.2%). Pathology was not obtained during data collection. The median age of onset was in the fourth decade of life. Of these individuals, 8 (47.1%) reported a family history of skin cancer. Moreover, 84 (38.2%) individuals reported a history of at least one skin biopsy due to suspicion for cancer. The median age of first biopsy was in the third decade of life. *Z*-test proportion analysis showed that this sample’s prevalence of skin cancer was significantly lower (*p* < 0.05) than the general population of 20%. Nearly 90% (*n* = 17) of those with a personal history of skin cancer reported having more than 20 AMN.

#### Vitiligo

Vitiligo was described as loss of skin coloring in blotches, and 13 (6.1%) reported a positive personal history. The median age of onset was in the second decade of life. Of this group, 3 (37.5%) reported a family history of vitiligo. The majority of vitiligo cases (*n* = 8, 61.5%) were untreated. Those who did report obtaining treatment most commonly reported the use of topical medication (*n* = 4, 30.8%) and light therapy (*n* = 2, 15.4%). *Z*-test proportion analysis demonstrated a significantly (*p* < 0.05) higher prevalence of vitiligo between this sample and the general population prevalence of approximately up to 2%.

#### Keloids

Keloids were defined as raised scars after an injury has healed. This was reported in 40.9% of respondents. The median age of onset was in the second decade of life. Of this group, 24 (40.7%) reported a family history of keloids. The most common body regions with keloids were the chest (*n* = 23, 26.1%) and back (*n* = 20, 22.7%). A z-test proportion analysis showed that this sample had a significantly (*p* < 0.05) higher prevalence of keloids compared to the general population of approximately 0.1% (12). Fischer’s exact analysis demonstrated a statistically significant (*p* < 0.05) association between an increased presence of keloids in individuals with both 45,X and 45,X/46,XX karyotypes.

#### Acne

Sixty-two (28.7%) respondents reported a personal history of acne. The median age of onset was during the second decade of life. Of these individuals, 26 (51.0%) reported a family history of acne. Of those with a history of acne, 46 (74.2%) reported a history of progesterone use. *Z*-test proportion analysis demonstrated a significantly (*p* < 0.05) lower prevalence of acne in this sample compared to the general population prevalence of 73.3%. See Table 4 for complete data on frequency, median age of onset, and positive family history.

### Skin effects of growth hormone therapy

Two-thirds (*n* = 144) of our sample reported a personal history of using GH therapy for treatment of short stature. Of those, 23.8% (*n* = 34) reported concerns about GH therapy and increased number of AMN, 19.4% (*n* = 28) about excess hair growth, and 23.8% (*n* = 34) with swelling. Of those with concerns about GH therapy and excess AMN, 5.9% (*n* = 2) reported a personal history of cancer. Of those with concerns with GH therapy and swelling, 55.9% (*n* = 19) reported a history of lymphedema.

### Quality-of-life impact

Overall, the mean QOL life impact score was 3.52 (SD, 3.42) corresponding to a small effect on patient lives according to the Dermatology Life Quality Index (DLQI) scoring system. An exploratory analysis with 2×2 contingency tables with chi-squared measures of association demonstrated that lymphedema, vitiligo, and xerosis cutis each were associated with a higher QOL impact score. T-tests confirmed these observations were statistically significant (*p* < 0.05) with xerosis cutis appearing to have the strongest relationship (*p* = 0.000). Linear regression further supported xerosis cutis having the largest relative contribution to quality-of-life impact.

Our sample was split into two groups (High quality of life impact and Low/Average quality of life impact) for further analysis. High QOL impact was defined as those with QOL impact scores two standard deviations above the mean, which corresponds to scores 11 or higher. Low/Average QOL impact was defined as 10 or lower. Of those with high QOL impact, t-tests showed a statistically significant associations (*p* < 0.05) with onychodystrophy, history of skin biopsy, and lymphedema.

Our cohort was then split into three subgroups (UTH registry, TSSUS, and Facebook) that was each defined by a separate avenue of data collection. Multivariate tests of means showed a significant difference (*p* < 0.05) in the quality-of-life impact scores between these three groups, where the TSSUS had a larger distribution of high QOL impact scores compared to the other two groups. Given this difference, a separate TSSUS-only exploratory analysis with 2×2 contingency tables with measures of association and t-tests was performed to investigate the validity of our inclusive data analysis. We found that the TSSUS had a statistically significant (*p* < 0.05) association between vitiligo and xerosis cutis with higher QOL impact scores. Lymphedema was not significantly associated in this group. A new relationship was found between >20 AMN and high QOL impact scores in this sub-cohort.

## Discussion

**Figure 1:**
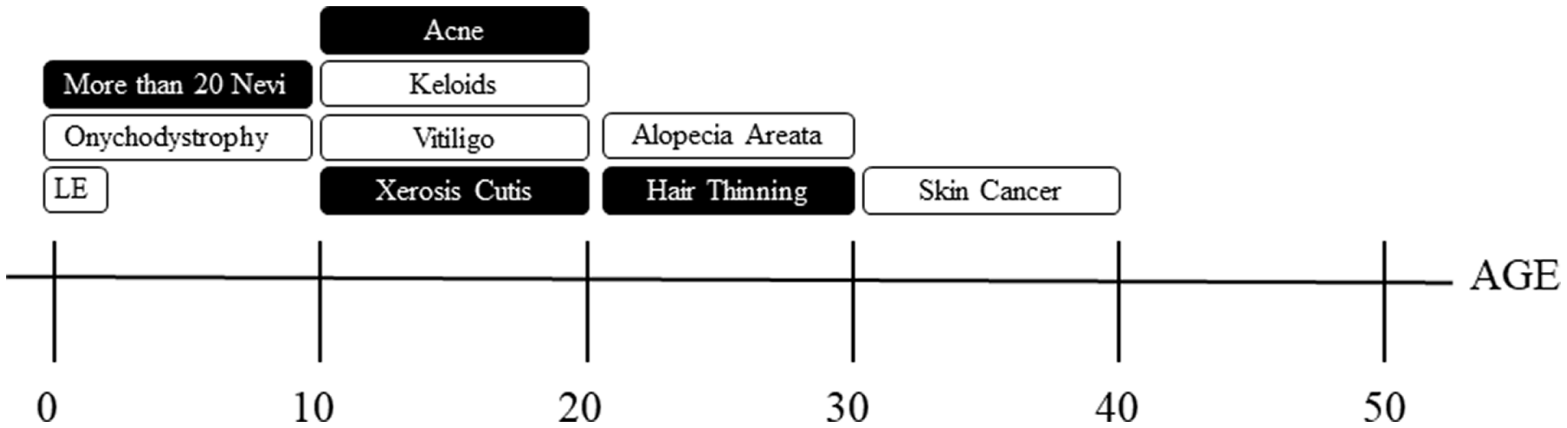
Timeline of dermatological manifestations. Shaded boxes indicate dermatological manifestations with over 50% positive family histories and those unshaded are those with less than 50% positive family histories. LE = lymphedema.

The overall goal of this study was to characterize the prevalence of self-reported dermatological concerns, treatment experiences, and impacts on quality of life in a large cohort of TS subjects. Our analysis in consistent with previous observations that dermatological manifestations are highly prevalent in TS (3, 13–16). We utilized this information to create a tailored timeline of TS-related dermatological manifestations visualized in Figure 1. Using this information in conjunction with the contribution of family history can be used to guide recommendations and management for dermatological care in the TS population.

Increased AMN have been commonly noted across TS literature in conjunction with theories and suggestions about a potential relationship with melanoma (13, 17). Our data showed a high prevalence of more than 20 AMN with an average onset in the first decade of life. However, the self-reported prevalence of skin cancer was approximately two times lower than the general population frequency of 20% (18, 19). The potential protective effect that TS has in skin cancer development is still under investigation (20).

Xerosis cutis was the most common skin concern reported in our cohort with the average age of onset being in the second decade of life which is much earlier than in the general population (21). One-third of respondents reported a personal history of lymphedema with an average age of diagnosis in the first decade highlighting a significantly lower prevalence than the previously reported two-thirds in the literature (22). This deviation is likely the result of recall bias as lymphedema is most common at the time of birth then typically resolves over time. Vitiligo was significantly four times more prevalent in our cohort compared to the general population (2%), but the mean onset in the second decade of life was similar to that of the general population (23). Consistent with previous literature, our sample confirmed that acne is much less common among TS girls. When acne is present, concomitant progesterone use was reported 75% of the time, indicating it may be a predictive factor.

Beyond TS, race and/or ethnicity plays a large role in risk of keloid development. Caucasians have a 0.1% risk for keloids and given that the majority of our cohort was Caucasian, we used 0.1% as a more appropriate general population risk for our cohort comparison (12). In this cohort, 40% had a personal history of keloids, most often starting in the second decade of life and located on the chest and/or back consistent with the literature (16). The central chest is a high-risk area for keloids, and since TS patients have many cardiovascular procedures, this may be a contributing factor the observed increased risk. Future investigations into the relationship between those with surgeries and keloids should be pursued.

In addition to prevalence data, we aimed to understand the frequency of growth hormone (GH) therapy usage, as well as side effect concerns among the TS population. Two-thirds of our cohort reported using GH therapy at some point in their lifespan (24). Of those, 23.8% had concerns about increased number of dermatological findings after initiating GH therapy. More research is needed to better understand the side effect profile of GH therapy in the TS population. Furthermore, there is a significant lack of treatment noted for common dermatology findings associated with TS. In our sample, 54.8% of those with lymphedema, 26.7%, of those with alopecia, and 61% of those with vitiligo currently do not utilize known available treatments (25). The lack of treatment could be attributed to numerous reasons, including infrequent referrals to dermatologist, but also concerns for side effects. This has not been adequately explored in our study and the TS population would benefit from future research in this realm.

The Dermatology Life Quality Index (DLQI) is a validated 10-point scale used to measure dermatology-specific quality-of-life impact scores. Our sample demonstrated a mean score of 3.52, which falls in the predetermined category of “small effect on quality of life.” More in-depth analysis demonstrated that three skin conditions had a statistically significant negative impact on quality-of-life; lymphedema, vitiligo, and xerosis cutis. Overall, xerosis cutis had the largest relative contribution to this detrimental effect on quality of life. This data suggests that xerosis cutis may be the primary predictor of individuals with TS with lower quality of life. When split into groups by high quality-of-life impact (≥ 11 score) and low/average quality of life impact (≤ 10 score); onychodystrophy, history of skin biopsy, and lymphedema were associated with a high QOL impact. This data suggests that a large proportion of TS-related skin conditions are significantly associated with a high impact on quality of life and require appropriate recognition by providers to mitigate this effect. Given the impact on quality of life, the 57.6% of our cohort who have a concern about their skin, and the 23.2% that current want a dermatological referral, there is need in this patient population to create and standardize dermatological care.

### Strengths and limitations

Robust strengths of our data include a large, multi-group analysis that included TS patients from a wide range of age groups and karyotype status. A clear limitation of this study’s methodology includes the self-report nature of our data. Self-report data includes risk for recall bias by respondents regarding their personal experience with the dermatological concerns queried in the survey. More specifically, dermatological conditions like lymphedema were unexpectedly underreported, which may indicate recall bias due to an average onset during infancy. In addition, the homogeneity of race and ethnicity (skewed to a Caucasian and Non-Hispanic Latina majority) introduces limitations to the interpretability of our data to these populations, where certain skin conditions may be at higher or lower prevalence based on ethnicity or race alone.

### Conclusion

Clinical guidelines recommend a dermatology referral at the time of diagnosis and continued follow-up as indicated (16). Our data reveal that skin conditions are highly prevalent in the TS population during the early decades of life and affirm utilizing these conditions in the TS diagnostic process, as well as emphasize the need for specialized dermatology referrals to address the detrimental impacts related to skin concerns on quality of life. Furthermore, our analysis provides evidence to corroborate a recommendation for routine dermatology care for patients with Turner syndrome in order to address significant physical and emotional impacts of this subset of clinical features.

## Data availability statement

The raw data supporting the conclusions of this article will be made available by the authors, without undue reservation.

## Ethics statement

The study protocol was approved by the Committee for the Protection of Human Subjects at the University of Texas Health Science Center at Houston. The studies were conducted in accordance with the local legislation and institutional requirements. Written informed consent for participation in this study was provided by the participants’ legal guardians/next of kin.

## Author contributions

DR-B, MM, SP, MR, MRi, and KR contribute to conception and design of the study. MM did data curation and lead statistical analysis and wrote the first draft of the manuscript while KR and MRo completed additional edits and formatting. All authors contributed to the article and approved the submitted version.

## Funding

This study was funded in part by a gift from the Turner Syndrome Society of the United States.

## Conflict of interest

The authors declare that the research was conducted in the absence of any commercial or financial relationships that could be construed as a potential conflict of interest.

## Publisher’s note

All claims expressed in this article are solely those of the authors and do not necessarily represent those of their affiliated organizations, or those of the publisher, the editors and the reviewers. Any product that may be evaluated in this article, or claim that may be made by its manufacturer, is not guaranteed or endorsed by the publisher.
