# Supplementary material for: Dermatological concerns for women and girls with turner syndrome
Source: Front Med (Lausanne). 2023 Sep 13;10:1235187. doi: 10.3389/fmed.2023.1235187 (PMC10533914; doi:10.3389/fmed.2023.1235187)
Supplement: Supplementary file 1 [file Data_Sheet_1.PDF]

Appendix A
Outline of Questionnaire for participant with Turner syndrome and/or parent

Rule out questions
Are you a member of a Turner syndrome related facebook group?
- Yes
- No

Are you a member of the UTHealth Turner syndrome research registry?
- Yes
- No

[If yes to either question, go on to next question]
[If no to both, skip next question]
Have you taken this skin questionnaire before via a facebook group or the UTHealth registry?
- Yes
- No

[If yes, end survey]

Demographics/Karyotype
1. I am:
   1. A person with Turner syndrome
   2. The parent or guardian of a person with Turner syndrome
   3. Someone else (other relative, friend)
2. Please select the age range you are within currently.
   1. 10-14
   2. 14-18
   3. 18-24
   4. 24-34
   5. 34-44
   6. 44-54
   7. 54+
3. What is your ethnicity?
   1. American Indian or Alaska Native
   2. Asian
   3. Black or African American
   4. Native Hawaiian or Other Pacific Islander
   5. White
4. How would you describe yourself?
   1. Hispanic or Latino
   2. Not Hispanic or Latino
5. Do you know your karyotype (how your chromosomes look—example: 45,X or one X chromosome)?
   1. Yes
   2. No

(If yes, go on)
(If no, skip to 6)

6. What is your karyotype (how your chromosomes look)?
    1. 45,X (one X chromosome in all cells)
    2. 45,X/46,XX (one X chromosome in some cells, two X chromosomes in some cells)
    3. 45,X/46,XY (one X chromosome in some cells, one X and one Y chromosome in some cells)
    4. Deletion Xp
    5. Isochromosome
    6. Mosaic with ring chromosome
    7. Mosaic with 47,XXX (some cells have three X chromosomes, some have two X chromosomes)
    8. Other mosaic with Y chromosome material
    9. Something else

Referral experience

7. Have you ever been referred to see a Dermatologist (skin doctor)?
    1. Yes
    2. No

(If no, skip to 11)
(If yes, go on)

8. Did a doctor who provides your Turner syndrome care make the referral?
    1. Yes
    2. No
9. Were you referred at the time of diagnosis of your Turner syndrome to see a Dermatologist or were you referred after a problem came up later?
    1. I was referred at my time of diagnosis.
    2. I was referred after a problem came up.
    3. Both
10. Did your doctor notice your skin concern during a doctor's visit or did you bring up your concern to your doctor?
    1. My doctor noticed a problem.
    2. I brought up my concerns to my doctor.
11. Are you currently concerned about your skin/hair/nails for any reason?
    1. Yes
    2. No
12. Would you like a referral to see a Dermatologist at this time?
    1. Yes
    2. No

Quality of Life Scale (Dermatology Life Quality Index (DLQI))

13. Over the last week, how itchy, painful, or stinging has your skin been?
    1. Very much
    2. A lot

    3. A little
    4. Not at all
14. Over the last week, how embarrassed or self conscious have you been because of your skin?
    1. Very much
    2. A lot
    3. A little
    4. Not at all
15. Over the last week, how much has your skin interfered with you going shopping or looking after your home or garden?
    1. Very much
    2. A lot
    3. A little
    4. Not at all
    5. Not relevant
16. Over the last week, how much has your skin influenced the clothes you wear?
    1. Very much
    2. A lot
    3. A little
    4. Not at all
    5. Not relevant
17. Over the last week, how much has your skin affected any social or leisure activities?
    1. Very much
    2. A lot
    3. A little
    4. Not at all
    5. Not relevant
18. Over the last week, how much has your skin made it difficult for you to do any sport?
    1. Very much
    2. A lot
    3. A little
    4. Not at all
    5. Not relevant
19. Over the last week, has your skin prevented you from working or studying?
    1. Yes
    2. No

(If no, go on)
(If yes, skip to 21)

20. If "no", over the last week how much has your skin been a problem at work or studying?
    1. A lot
    2. A little
    3. Not at all

21. Over the last week, how much has your skin created problems with your partner or any of your close friends or relatives?
    1. Very much
    2. A lot
    3. A little
    4. Not at all
    5. Not relevant
22. OVer the last week, how much has your skin caused any sexual difficulties?
    1. Very much
    2. A lot
    3. A little
    4. Not at all
    5. Not relevant
23. Over the last week, how much of a problem has the treatment for your skin been, for example by making your home messy, or by taking up time?
    1. Very much
    2. A lot
    3. A little
    4. Not at all
    5. Not relevant

Skin concerns

(For each of the following from 7-33, a "yes" answer will reflex to asking what **age the concern onset and medication history**)

24. Do you ever develop dry, flaky or scaly skin?
    1. Yes
    2. No
25. Have you heard of lymphedema (excess fluid collecting in tissues causing swelling)?
    1. Yes
    2. No
26. Has your physician/health caver provider ever talked with you about lymphedema?
    1. Yes
    2. No
27. Have you ever had lymphedema?
    1. Yes
    2. No

(If no, skip to 39)

28. At what age were you diagnosed with lymphedema? ___________
29. How long did you have/have you had lymphedema?
    1. <1 year
    2. 1-5 years
    3. 6-10 years
    4. 11+ years
30. Has your lymphedema resolved?

    1. Yes
    2. No
31. Have you ever developed an infection of the skin or tissue that has lymphedema?
    1. Yes
    2. No
32. If yes to 32, at what age did you develop the infection? ____
33. What area(s) of the body did you experience lymphedema? Check all that apply.
    1. Arms
    2. Legs
    3. Torso (mid-section of the body)
    4. Neck or head
    5. Genitals
    6. Other
34. Did you experience abnormal nail growth due to the lymphedema?
    1. Yes
    2. No
35. Have you experienced difficulty walking or gait problems due to swelling?
    1. Yes
    2. No
36. Have you experienced difficulty dressing, bathing or doing activities at home like folding clothes, washing dishes or opening jars because of arm or hand swelling?
    1. Yes
    2. No
37. What treatment(s) did you receive for your lymphedema?
    1. Manual lymphatic drainage/specialized massage.
    2. Compression garment or wrap.
    3. Pneumatic compression therapy.
    4. Other
    5. None
38. Have you been able to fully comply with your prescribed lymphedema treatments at home?
    1. Yes
    2. No
39. Have you ever experienced hair loss or thinning of your hair?
    1. Yes
    2. No
40. Have you ever had small, pitted, abnormally shaped and/or painful nails?
    1. Yes
    2. No
41. Do you have more than 20 moles on your skin?
    1. Yes
    2. No
42. Do you have raised red marks and/or patches on your skin?

   1. Yes
   2. No

43. Have you ever had any skin lesions biopsied due to suspicion for cancer?
   1. Yes
   2. No

44. Have you ever been diagnosed with skin cancer?
   1. Yes
   2. No

(If yes, go on)
(If no, skip to 40)

45. What area(s) of the body did you have skin cancer? Check all that apply.
   1. Face
   2. Head
   3. Neck
   4. Hands
   5. Chest and back
   6. Legs
   7. Hands, feet, or nail beds
   8. Other

46. Was the affected area sun exposed or not sun exposed?
   1. Sun exposed
   2. Not sun exposed

47. Have you ever had vitiligo (loss of skin coloring in blotches)?
   1. Yes
   2. No

(If yes, go on)
(If no, skip to 43)

48. What area of the body did you <u>first</u> experience vitiligo?
   1. Face
   2. Neck
   3. Armpits
   4. Elbows
   5. Genitalia
   6. Hands
   7. Knees
   8. Other

49. What treatment(s) did your doctor use to treat your vitiligo? Check all that apply.
   1. Medications (oral)
   2. Medications (topical)
   3. Light therapy
   4. Combining psoralen and light therapy
   5. Removing the remaining color (depigmentation)
   6. Surgery (grafting or skin transplant)

7. Other
8. None

50. Have you ever had alopecia (sudden hair loss resulting in baldness)?
    1. Yes
    2. No

(If yes, go on)
(If no, skip to 46)

51. Have you experienced alopecia in any other body region besides your scalp (ex. Face, arms, legs)?
    1. Yes
    2. No
52. What treatment(s) did your doctor use to treat your alopecia? Check all that apply.
    1. Topical corticosteroids
    2. Rogaine
    3. Injections of corticosteroids
    4. Contact immunotherapy
    5. Methotrexate medication
    6. Other oral medications
    7. Cosmetic replacements (i.e. wigs, makeup)
    8. Other
    9. None
53. Have you ever developed a keloid (raised scar after an injury has healed)?
    1. Yes
    2. No

(If yes, go on)
(If no, skip to 48)

54. What area(s) of your body did you experience keloids? Check all that apply.
    1. Neck
    2. Shoulders
    3. Chest
    4. Back
    5. Ears
    6. Other
55. Have you ever noticed abnormal scarring or problems with delayed wound healing?
    1. Yes
    2. No
56. Have you ever had problems with acne?
    1. Yes
    2. No

(If yes, go on)
(If no, skip to 51)

57. Have you ever been prescribed progesterone hormone therapy?
    1. Yes

2. No
58. Have you ever taken growth hormone medications?
    1. Yes
    2. No

(If yes, go on)
(If no, skip to 55)

59. Do you have any concerns about dermatological side effects of medications (ex. growth hormone) and increased number of skin moles?
    1. Yes
    2. No
60. Do you have any concerns about dermatological side effects of medications (ex. growth hormone) and excess body hair growth?
    1. Yes
    2. No
61. Do you have any concerns about dermatological side effects of medications (ex. Growth hormone) and swelling of the hands and/or feet?
    1. Yes
    2. No
62. Do you have any other dermatological concerns other than those listed in this survey? List them here.

Family history

63. Please check all skin concerns that you have a family history of. Family history is defined as having one or more relatives (mother, father, sister, brother) with a specific medical problem.
    1. Dry, flaky or scaly skin
    2. Lymphedema (excess fluid collecting in tissues causing swelling)
    3. Skin infections due to lymphedema
    4. Hair loss or thinning of the hair
    5. Small, pitted, abnormally shaped and/or painful nails
    6. 20+ moles
    7. Raised red marks and/or patches on the skin
    8. Skin biopsies due to suspicion of skin cancer
    9. Melanoma (skin cancer)
    10. Vitiligo (loss of skin coloring in blotches)
    11. Alopecia (sudden hair loss resulting in baldness)
    12. Keloids (raised scar after an injury has healed)
    13. Abnormal scarring or delayed wound healing
    14. Problems with acne
